# Malaria‐Associated Secondary Hematophagocytic Lymphohistocytosis—A Case Report and Comprehensive Literature Review (sHLH)

**DOI:** 10.1002/ccr3.73434

**Published:** 2026-08-30

**Authors:** Indresh Yadav, Sulav Kumar Jha, Sweta Singh, Dipesh Kumar Mandal, Saurav Jha

**Affiliations:** ^1^ Northwell Vassar Brothers Medical Centre Poughkeepsie New York USA; ^2^ Nepal Manipal College of Medical Sciences Pokhara Nepal; ^3^ Department of Medicine B.P. Koirala Institute of Health Sciences Dharan Nepal; ^4^ Patan Academy of Health Sciences Kathmandu Nepal

**Keywords:** case report, hemophagocytic lymphohistiocytosis, malaria, plasmodium

## Abstract

Malaria‐associated secondary HLH, though rare, is often overlooked. Clinicians should maintain a high‐index of suspicion in travelers returning from malaria‐endemic regions who develop severe to rapidly worsening inflammatory illness, as early recognition is critical for survival.

## 1 Introduction

Hemophagocytic lymphohistocytosis (HLH) is a life‐threatening syndrome of pathological immune activation characterized by fever, cytopenias, hyperferritinemia, coagulopathy, and organ dysfunction. Diagnostic criteria were originally developed for pediatric HLH and are often applied to adults; the HScore is a commonly used adult‐oriented diagnostic approach [1, 2].

In adults HLH frequently overlaps clinically with severe sepsis and other hyperinflammatory states, making timely recognition difficult [3, 4]. Very high ferritin supports the diagnosis, but is not specific in adults; additional immune activation markers (e.g., soluble IL‐2 receptor) and overall clinical context improve diagnostic confidence [5, 6, 7].

Malaria is an uncommon but increasingly reported trigger of secondary HLH (sHLH). Recent multicenter data and literature reviews suggest that malaria‐associated HLH is rare and reported in < 1% of malaria cases in high income settings, but may be underdiagnosed and can be fatal without prompt recognition and individualized immunomodulatory therapy in selected patients.

## 2 Case Report

### 2.1 Case History and Examination

A 70‐year‐old woman with no known prior chronic illnesses arrived as a transfer for urgent evaluation after a rapidly worsening illness. She had returned only a few days earlier from a bird‐watching trip to Papua New Guinea, during which she stayed in hotels and camped outdoors. She reported multiple mosquito bites and had not taken malarial prophylaxis.

Soon after her return, she developed a high‐grade fever (up to 104.2°F), large volume watery diarrhea, marked weakness, and progressive shortness of breath at rest. On examination, the patient was ill‐looking and exhibited the signs of dehydration but lacked pertinent findings such as pallor, icterus, cyanosis, clubbing, lymphadenopathy, or edema. On further assessment at the hospital, she was tachycardic (123 beats per minute), tachypneic (26 breaths per minute with the use of accessory muscles), and hypotensive, requiring vasopressor support despite adequate fluid resuscitation.

### 2.2 Differential Diagnosis/Investigations/Treatment

Initial laboratory investigations revealed marked leukocytosis with bandemia, severe thrombocytopenia (platelets ~17,000/μL), elevated liver transaminases, acute kidney injury, hyponatremia, and coagulopathy. A peripheral blood smear demonstrated *Plasmodium falciparum* with high parasitemia, confirming severe malaria. She was empirically commenced on broad‐spectrum antimicrobials for presumed sepsis (IV vancomycin, doxycycline, atovaquone, piperacillin‐tazobactum) prior to antimalarial therapy (Artemether–Lumefantrine).

Despite appropriate treatment, her condition deteriorated. She became increasingly dyspnoeic at rest, lethargic, and hemodynamically unstable, with evolving multiorgan dysfunction. The patient's condition continued to deteriorate and she was admitted to ICU for sepsis and multiorgan failure secondary to severe malaria.

At the time of ICU admission, the working diagnosis included severe malaria with parasitemia complicated by multiorgan dysfunction syndrome, severe thrombocytopenia, sepsis with septic shock, sepsis‐associated acute kidney injury, hemolytic anemia, acute infective diarrhea with isolated organisms, and acute hypoxic respiratory failure.

Her laboratory investigations were markedly abnormal. The patient had leucocytosis with a white blood cell count of 13.3 × 10^9^/L (reference range: 4.5–11.0 × 10^9^/L) and significant bandemia of 40% (reference range: < 5%), with a hemoglobin level of 12.7 g/dL (reference range: 12.0–15.5 g/dL) which was within normal range. She was noted to have severe thrombocytopenia, with platelet counts of 17,000/μL (reference range: 150,000–450,000/μL). Renal function was impaired, with a serum creatinine of 1.86 mg/dL, which subsequently rose to 2.26 mg/dL (reference range: 0.6–1.1 mg/dL), consistent with acute kidney injury. Liver enzymes were also significantly elevated, with alanine aminotransferase (ALT) ranging from 130 to 169 U/L (reference range: 7–55 U/L), and aspartate aminotransferase (AST) ranging from 263 to 315 U/L (reference range: 8–48 U/L). Coagulation studies demonstrated prolongation of the international normalized ratio (INR) to 1.7 (reference range: 0.8–1.1), and serum electrolytes revealed hyponatremia with sodium level of 123 mmol/L (reference range: 135–145 mmol/L).

Although serial blood smears demonstrated a decline in malarial parasitemia to approximately 2%, her clinical status paradoxically worsened, prompting further evaluation for secondary inflammatory and hematological complications. Further evaluation revealed laboratory features suggestive of secondary hemophagocytic lymphohistiocytosis (HLH). Serum fibrinogen was reduced to 158 mg/dL (reference range: 200–400 mg/dL), lactate dehydrogenase (LDH) was markedly elevated to 1734 U/L (reference range: 140–230 U/L), and serum ferritin was profoundly elevated at 76,179 ng/mL (reference range: 11–307 ng/mL). Soluble IL‐2 receptor levels were initially pending and later returned markedly elevated at 35,564 U/mL (reference range: 175.3–858.2 U/mL), further supporting the diagnosis.

Given the high clinical suspicion for secondary hemophagocytic lymphohistiocytosis (HLH), hematology recommended completion of intravenous immunoglobulin (IVIG) at 40 g daily for 2 days, along with high dose intravenous methyl‐prednisolone (solu‐medrol) 500 mg for two doses and anakinra at an initial dose of 400 mg. Close laboratory monitoring was instituted including daily fibrinogen, ferritin, LDH, coagulation profile, complete blood count (CBC), and comprehensive metabolic panel (CMP).

Following initiation of HLH‐directed therapy, intravenous methylprednisolone was transitioned to intravenous hydrocortisone. Despite initial treatment, fibrinogen levels continued to decline to 89 mg/dL, necessitating administration of cryoprecipitate. The patient required multiple platelet transfusions due to persistent severe thrombocytopenia. The patient developed persistent diarrhea, with stool studies positive for enteropathogenic *Escherichia coli* and norovirus; *Clostridioides difficile* testing was negative. Her ICU course was further complicated by episodes of bradycardia and hypotension, which were refractory to fluid resuscitation and required dopamine infusion, later transitioned to epinephrine, resulting in hemodynamic improvement.

Transthoracic echocardiography demonstrated preserved left ventricular ejection fraction, with moderate mitral regurgitation and mild‐to‐moderate tricuspid regurgitation. Thyroid‐stimulating hormone levels were normal, and lyme serology was negative. She completed a full course of artemether–lumefantrine for malaria. Following transfer to the medical floor, anakinra was continued at 100 mg once daily, and steroid therapy was switched to oral prednisolone 40 mg daily. Because of insurance barriers, anakinra could not be continued after discharge, and the patient was discharged on corticosteroids (prednisone 60 mg daily).

**FIGURE 1:**
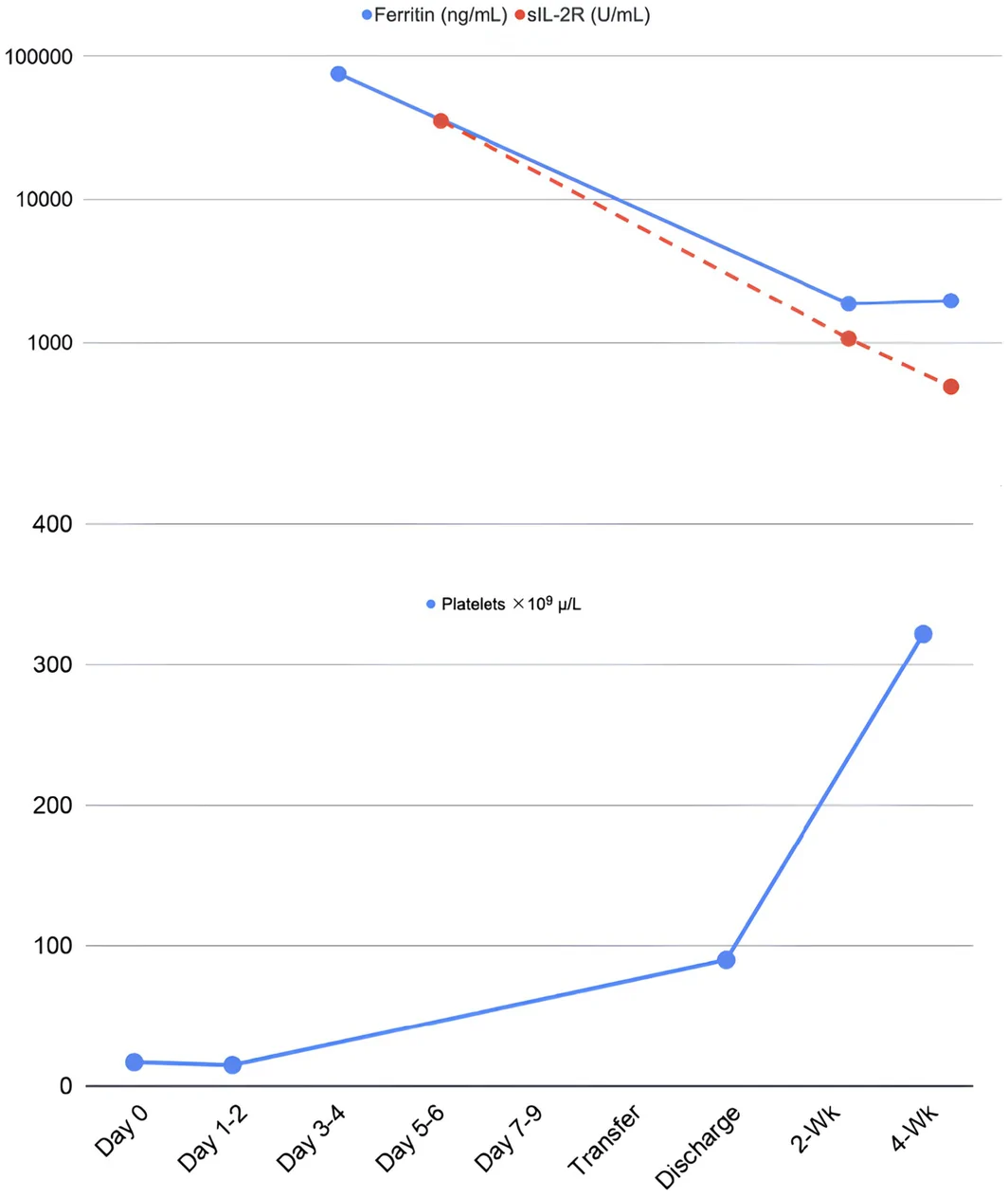
Clinical timeline showing hyperinflammation resolution and hematologic recovery.

At the time of discharge, laboratory studies demonstrated marked improvement with WBC count of 3.2 × 10^9^/L, hemoglobin of 7.8 g/dL, platelet count improved to 90,000/μL, fibrinogen 143 mg/dL, BUN 42 mg/dL, creatinine 1.16 mg/dL, and normal electrolytes. Inflammatory markers had markedly improved, with LDH 309 U/L and ferritin 1868 ng/mL. To aid comprehension of this complex clinical course, a chronological timeline showing hyperinflammation resolution and hematologic recovery is shown in Figure 1.

### 2.3 Outcome/Follow‐Up

At 2‐week follow‐up, ferritin was 1961 ng/L and IL‐2 receptor levels had decreased to 1062.2 U/mL. At subsequent follow‐up 2 weeks later, laboratory values showed near complete resolution of inflammation, with IL‐2 receptor 491 U/mL, and platelet count recovered to 322,000/μL. Corticosteroids were tapered to 20 mg daily, and the patient reported marked symptomatic improvement with no residual complaints.

## 3 Discussion

Hematophagocytic lymphohistocytosis (HLH), also known by hematophagocytic syndrome, is caused by a defect in inflammatory signal that results in a setting of congenital or acquired defective natural killer (NK) T Cell function in the cytotoxic pathway [8] and falls under the umbrella concept of cytokine storm syndrome (CSS) [9]. Primary HLH, also known as familial HLH, is a genetic disorder that usually presents in infancy or early childhood, which results from an inherited mutation affecting cytotoxic function of natural killer (NK cells) and CD8+ T lymphocytes and follows an autosomal recessive inheritance pattern [10]. Secondary HLH (sHLH) is an acquired condition that can occur at any age and is triggered by strong immune stimulation, preceded by precipitating factors such as infection following EBV, malignancies (particularly hematological causes), autoimmune diseases, and immunosuppressive states [10]. Although primary and secondary HLH differ in etiology, both share a common pathophysiological pathway of uncontrolled immune activation, making prompt diagnosis and treatment essential for patient survival.

Malaria associated with secondary hematophagocytic lymphohistocytosis (sHLH) is a rare complication, but one that is probably missed more often than it is recognized. A multicenter study from Germany covering the period of 2015–2022, combined with a systematic review of literature, found sHLH in approximately 0.34% of treated malaria cases [11]. Most reported cases were linked to *Plasmodium vivax* and *P. falciparum*, and the overall mortality across published reports was about 5% [11].

Diagnosis of HLH is challenging because initial symptoms are often non‐specific, yet prompt recognition is critical due to the rapidly progressive nature and high mortality of the disease. In clinical practice, diagnosis most commonly relies on the HLH‐2004 criteria and the HScore, although applying these tools in critically ill adults is frequently limited by incomplete or unavailable data [9]. Measurement of soluble IL‐2 receptors (sIL‐2R, CD25), a marker of T‐cell activation, has shown a strong diagnostic utility. In adult populations, values exceeding 10,000 U/mL have been described as highly specific for HLH [5, 6, 7].

Ferritin should be interpreted with caution rather than treated as a definitive marker. Although levels ≥ 10,000 ng/mL are strongly suggestive of HLH in pediatric populations, adult data show that extreme hyperferritinemia may also be seen in conditions such as severe hepatic injury, renal failure, infections, and malignancies [5, 6]. Ferritin levels rise markedly in diseases such as HLH because activated macrophages, which proliferate and actively phagocytose erythrocytes and accumulate large amounts of intracellular ferritin and release it into the circulation, make serum ferritin a reflection of macrophage activation and disease activity in histiocytic proliferative and hyperinflammatory conditions [8]. Therefore, evaluating trends over time and integrating supportive findings such as hypofibrinogenemia, cytopenias, and elevated soluble IL‐2 receptor levels enhances diagnostic accuracy.

Treatment of malaria‐associated secondary HLH centers on two priorities: prompt eradication of the parasite and tailored immunomodulatory therapy when excessive inflammation compromises organ function [12]. Both international and national guidelines recommend intravenous or intramuscular artesunate as the treatment of choice for severe malaria, with subsequent completion of the artemisinin‐based combination regimen once the patient can tolerate oral medication [13, 14].

For adult sHLH, expert recommendations emphasize treating the trigger, supportive ICU care, and early immunosuppression for patients with progressive organ dysfunction [15]. Common approaches include high‐dose corticosteroid and IVIG; etoposide‐based regimens are considered in selected high‐risk presentations (EBV‐associated HLH or malignancy‐associated HLH) [15].

IL‐1 blockade with anakinra has emerged as a favored option in hyperinflammatory states where infection is present or suspected because it targets a key cytokine axis while avoiding the myelotoxicity of etoposide [16]. Evidence supporting this approach includes signals of benefit in sepsis patients with macrophage activation‐like features. Furthermore, there is growing literature supporting the safety and efficacy of anakinra specifically in infection‐associated secondary adult HLH, alongside its broader established application across other secondary HLH triggers, such as high‐risk hematologic settings [17].

While the importance of IL‐1 as a key cytokine is well‐established, there is growing evidence for the utility of CXCL9 and IL‐18 as biomarkers for predicting treatment response and evaluating efficacy in HLH [18]. In our case, these specific markers were not obtained due to lack of widespread rapid availability at our institution; however, it is important to recognize their utility in assessing interferon‐gamma and inflammasome activation, especially while evaluating targeted therapies such as Janus kinase inhibitors (JAKi). Many reported malaria‐associated sHLH cases improve with anti‐malarial therapy alone. Adjunctive immunomodulation (steroids, IVIG, and less commonly targeted cytokine inhibition or JAK inhibition) is typically reserved for patients with persistent shock, cytopenias, or coagulopathy [16, 17, 19]. To contextualize this case, selected published reports of malaria‐associated secondary HLH are summarized in Table 1, illustrating a range of plasmodium species and therapeutic strategies [11, 20, 21, 22, 23].

**TABLE 1:** Selected published reports of malaria‐associated secondary HLH.

References	Species/Settings	Key sHLH features	HLH‐directed therapy	Outcome	References
Muthu et al.	*P. vivax*/mixed (India)	ARDS, pancytopenia, ferritin increased, fibrinogen decreased	IVIG+ Antimalarials	Recovered	[20]
Orth et al.	Multispecies (Germany)	sHLH in 0.34% of malaria, review of 80 cases	Varied, 41% received sHLH therapy	CFR 5%	[11]
Zhou and Duan	Malaria associated sHLH	Cytopenias, increased ferritin, organ dysfunction	Immunomodulation	Recovered	[21]
Fuchs et al.	Imported *P. falciparum*	sHLH with systemic hyperinflammation	Ruxolitinib+ supportive care	Recovered	[22]
Ullah et al.	*P. vivax*	Fever, cytopenias, increased ferritin	Steroids	Recovered	[23]

Abbreviations: ARDS, acute respiratory distress syndrome; CFR, case fatality rate; IVIG, intravenous immunoglobulin; *P. falciparum*, *Plasmodium falciparum*; *P. vivax*, *Plasmodium vivax*; sHLH, secondary hemophagocytic lymphohistiocytosis.

This case highlights a rare but important complication of severe *P. falciparum* malaria, namely secondary hemophagocytic lymphohistiocytosis (sHLH) and emphasizes the need for early recognition when patients worsen despite improving parasitemia. A major strength of this report is the detailed description of the clinical course, serial laboratory evolution, and response to combined antimalarial and HLH‐directed therapy, which may help clinicians facing similar diagnostic uncertainty. However, this report has several limitations. As a single‐patient observation, its findings cannot be generalized broadly, and some treatment decisions were individualized based on the patient's rapidly evolving clinical condition rather than standardized protocols. In addition, although the diagnosis of sHLH was strongly supported by the overall clinical picture and markedly elevated inflammatory markers, diagnostic assessment in critically ill adults can be challenging because HLH criteria are often applied in settings with overlapping sepsis and multiorgan dysfunction.

## 4 Conclusion

Our case illustrates a diagnostic trap; severe falciparum malaria can closely resemble septic shock, and malaria‐associated sHLH can further amplify the inflammatory phenotype. Clinicians must remain vigilant and maintain a high index of suspicion for secondary HLH in patients living or returning from malaria‐endemic areas. Most importantly, when a patient worsens despite improving parasitemia, clinicians should consider sHLH, particularly when cytopenias and coagulopathy are prominent.

## Author Contributions

**Indresh Yadav:** conceptualization, investigation, writing – review and editing. **Dipesh Kumar Mandal:** writing – review and editing. **Saurav Jha:** writing – review and editing. **Sweta Singh:** conceptualization, writing – review and editing. **Sulav Kumar Jha:** conceptualization, writing – original draft, writing – review and editing.

## Funding

The authors have nothing to report.

## Consent

Written informed consent was obtained from the patient for publication of this report and accompanying images in accordance with the journal's patient consent policy.

## Conflicts of Interest

The authors declare no conflicts of interest.

## Data Availability

Data will be provided by the corresponding author upon reasonable request.

## References

[1] L. Fardet , L. Galicier , O. Lambotte , et al., “Development and Validation of the HScore, a Score for the Diagnosis of Reactive Hemophagocytic Syndrome,” Arthritis & Rhematology 66, no. 9 (2014): 2613–2620, 10.1002/art.38690. 24782338

[2] J. Henter , A. Horne , M. Aricó , et al., “HLH‐2004: Diagnostic and Therapeutic Guidelines for Hemophagocytic Lymphohistiocytosis,” Pediatric Blood & Cancer 48, no. 2 (2007): 124–131, 10.1002/pbc.21039. 16937360

[3] R. A. Raschke and R. Garcia‐Orr , “Hemophagocytic Lymphohistiocytosis,” Chest 140, no. 4 (2011): 933–938, 10.1378/chest.11-0619. 21737492

[4] K. Bauchmuller , J. J. Manson , R. Tattersall , et al., “Haemophagocytic Lymphohistiocytosis in Adult Critical Care,” Journal of the Intensive Care Society 21, no. 3 (2020): 256–268, 10.1177/1751143719893865. 32782466 PMC7401439

[5] A. Hayden , M. Lin , S. Park , et al., “Soluble Interleukin‐2 Receptor Is a Sensitive Diagnostic Test in Adult HLH,” Blood Advances 1, no. 26 (2017): 2529–2534, 10.1182/bloodadvances.2017012310. 29296904 PMC5728644

[6] A. M. Schram , F. Campigotto , A. Mullally , et al., “Marked Hyperferritinemia Does Not Predict for HLH in the Adult Population,” Blood 125, no. 10 (2015): 1548–1552, 10.1182/blood-2014-10-602607. 25573993

[7] K. Sackett , M. Cunderlik , N. Sahni , A. A. Killeen , and A. P. J. Olson , “Extreme Hyperferritinemia: Causes and Impact on Diagnostic Reasoning,” American Journal of Clinical Pathology 145, no. 5 (2016): 646–650, 10.1093/ajcp/aqw053. 27247369

[8] N. Verma , J. Chakraverty , P. Baweja , A. Girotra , L. Chatterjee , and M. Chugh , “Extremely High Ferritinemia Associated With Haemophagocytic Lympho Histiocytosis (HLH),” Indian Journal of Clinical Biochemistry 32, no. 1 (2017): 117–120, 10.1007/s12291-016-0559-8. 28149024 PMC5247359

[9] M. Goubran and L. Chen , “Hemophagocytic Lymphohistiocytosis and Other Cytokine Storm Syndromes in Adults,” Canadian Hematology Today 4 (2025): 37–46, 10.58931/cht.2025.4166.

[10] G. E. Janka , “Hemophagocytic Syndromes,” Blood Reviews 21, no. 5 (2007): 245–253, 10.1016/j.blre.2007.05.001. 17590250

[11] H. M. Orth , D. Wiemer , S. Schneitler , et al., “Hemophagocytic Lymphohistiocytosis—How Common and How Severe Is It as a Complication of Malaria? Retrospective Case Series and Review of the Literature,” Infection 52, no. 2 (2024): 471–482, 10.1007/s15010-023-02104-w. 37875775 PMC10955030

[12] World Health Organization , WHO Guidelines for Malaria (World Health Organization, 2025), 10.2471/B09514.

[13] A. M. Dondorp , C. I. Fanello , I. C. Hendriksen , et al., “Artesunate Versus Quinine in the Treatment of Severe Falciparum Malaria in African Children (AQUAMAT): An Open‐Label, Randomised Trial,” Lancet 376, no. 9753 (2010): 1647–1657, 10.1016/S0140-6736(10)61924-1. 21062666 PMC3033534

[14] E. B. Yunus , “Artesunate Versus Quinine for Treatment of Severe Falciparum Malaria: A Randomised Trial,” Lancet 366, no. 9487 (2005): 717–725, 10.1016/S0140-6736(05)67176-0. 16125588

[15] P. La Rosée , A. Horne , M. Hines , et al., “Recommendations for the Management of Hemophagocytic Lymphohistiocytosis in Adults,” Blood 133, no. 23 (2019): 2465–2477, 10.1182/blood.2018894618. 30992265

[16] P. Mehta , R. Q. Cron , J. Hartwell , J. J. Manson , and R. S. Tattersall , “Silencing the Cytokine Storm: The Use of Intravenous Anakinra in Haemophagocytic Lymphohistiocytosis or Macrophage Activation Syndrome,” Lancet Rheumatology 2, no. 6 (2020): e358–e367, 10.1016/S2665-9913(20)30096-5. 32373790 PMC7198216

[17] B. Shakoory , J. A. Carcillo , W. W. Chatham , et al., “Interleukin‐1 Receptor Blockade Is Associated With Reduced Mortality in Sepsis Patients With Features of Macrophage Activation Syndrome: Reanalysis of a Prior Phase III Trial*,” Critical Care Medicine 44, no. 2 (2016): 275–281, 10.1097/CCM.0000000000001402. 26584195 PMC5378312

[18] X. Lan , N. Wei , J. Wang , and Z. Wang , “CXCL9 and IL‐18: Potential Biomarkers for Efficacy Evaluation in Refractory Hemophagocytic Lymphohistiocytosis Treated With RED (Ruxolitinib, Emapalumab and Dexamethasone),” Annals of Hematology 104, no. 3 (2025): 1459–1469, 10.1007/s00277-025-06336-8. 40186662 PMC12031756

[19] H. Al‐Yousuf , J. O'Nions , A. J. Wilson , S. Gohil , J. J. Manson , and E. M. Payne , “Safety and Efficacy of Anakinra in Hemophagocytic Lymphohistiocytosis Associated With Acute Leukemia,” Haematologica 109 (2024): 1947–1950, 10.3324/haematol.2023.283879. 38299608 PMC11141663

[20] V. Muthu , S. Dhooria , I. S. Sehgal , R. Agarwal , D. Behera , and N. Varma , “Malaria‐Associated Secondary Haemophagocytic Lymphohistiocytosis: Report of Two Cases & a Review of Literature,” Indian Journal of Medical Research 145, no. 3 (2017): 399–404, 10.4103/ijmr.IJMR_740_15. 28749405 PMC5555071

[21] X. Zhou and M. L. Duan , “Malaria‐Associated Secondary Hemophagocytic Lymphohistiocytosis: A Case Report,” World Journal of Clinical Cases 9, no. 22 (2021): 6403–6409, 10.12998/wjcc.v9.i22.6403. 34435005 PMC8362573

[22] A. Fuchs , H. M. Orth , U. Germing , et al., “Falciparum Malaria‐Induced Secondary Hemophagocytic Lymphohistiocytosis Successfully Treated With Ruxolitinib,” International Journal of Infectious Diseases 100 (2020): 382–385, 10.1016/j.ijid.2020.07.062. 32777582

[23] W. Ullah , H. M. A. Abdullah , S. Qadir , and M. A. Shahzad , “Haemophagocytic Lymphohistiocytosis (HLH): A Rare but Potentially Fatal Association With *Plasmodium vivax* Malaria,” BML Case Reports 2016 (2016): bcr2016215366, 10.1136/bcr-2016-215366. PMC4932401 27298293

